# Quantitative and Qualitative Analysis of Surface Modified Cellulose Utilizing TGA-MS

**DOI:** 10.3390/ma9060415

**Published:** 2016-05-25

**Authors:** Daniel Loof, Matthias Hiller, Hartmut Oschkinat, Katharina Koschek

**Affiliations:** 1 Adhesive Bonding Technology and Surfaces, Fraunhofer Institute for Manufacturing Technology and Advanced Materials IFAM, Wiener Strasse 12, Bremen 28359, Germany; daniel.loof@kabelmail.de; 2 Department of Biology/Chemistry (FB2), University of Bremen, Leobener Strasse NW2C, Bremen 28359, Germany; 3 Department NMR-supported Structural Biology, Leibniz-Institut für Molekulare Pharmakologie, Robert-Rössle-Strasse 10, Berlin 13125, Germany; hiller@fmp-berlin.de (M.H.); oschkinat@fmp-berlin.de (H.O.)

**Keywords:** thermogravimetric analysis, evolved gas analysis, silane treatment, microcrystalline cellulose, thiol functionalization

## Abstract

With the aim to enhance interfacial adhesion of a hydrophobic polymer matrix and cellulosic fibers and fillers, chemical surface modifications with silane coupling agents are performed. Thermogravimetric analysis (TGA) could be used to determine the degree of surface functionalization. However, similar thermal properties of treated and untreated cellulose hamper a precise determination of silane loading. This contribution deals with quantitative determination of silane loading combining both TGA and elemental analysis. Firstly, silane modified celluloses were studied by FT-IR, Raman, solid state NMR spectroscopy, and polarized light microscopy in order to determine functional groups and to study the impact of chemical treatment on cellulose morphology. Secondly, thermal stability and pyrolysis processes were studied by TG-MS analysis. In order to determine the exact silane loading, the mass percentages of the appropriate elements were quantified by elemental analysis and correlated with the charred residues determined by TGA yielding a linear dependency. With that correlation, it was possible to determine silane loadings for additional samples utilizing simple TGA measurements. The main advantage of that approach is that only one calibration is necessary for routine analyses of further samples and TGA-MS coupling gives additional information on thermal stability and pyrolysis routes, simultaneously.

## 1. Introduction

Fiber reinforced plastics (FRP) are often used for light weight constructions due to their high weight-specific stiffness as well as strength. Besides carbon and glass, lignocellulosic fibers such as flax, sisal, jute, ramie, hemp, kenaf, and some potential new fibers such as the cellulosic fiber extracted from *Passiflora foetida* constitute an interesting sustainable alternative to traditional synthetic fibers due to their low cost, low density, ecofriendly nature, and availability [1,2,3,4,5,6]. Natural fibers, however, exhibit hydrophilic properties, which result in poor wetting when processed with hydrophobic polymer matrices [7,8]. This results in insufficient stress transfer from matrix to the reinforcing fibers restricting the mechanical properties of natural fiber reinforced composites (NFC). In order to enhance the interfacial adhesion, natural fibers are generally modified by physical methods such as plasma and corona discharge [9,10,11] as well as chemical treatments e.g., esterification, etherification, or oxidation [12,13,14,15,16,17,18]. A widely applied chemical method is the treatment of cellulose with silane coupling agents [19,20,21,22]. Surface silylation has an impact on the physicochemical properties of lignocellulosic fibers altering, e.g., moisture absorption, chemical resistance, thermal degradation, surface hydrophilicity, and improving the mechanical properties of cellulosic composites [23,24,25,26,27]. There are different approaches how to quantify the grafted silane amount. Among others, elemental analysis (EA) delivers exact chemical compositions determining mass percentages of each present element [28]. It requires, however, very pure compounds free of impurities, which would falsify the result. A further approach to determine cellulose loading with silane and other functional groups is thermogravimetric analysis (TGA) [26,29]. TGA monitors chemical reactions and physical conversions taking place during the heating process by detecting mass change as a function of temperature and time and the charred residue. That gives information regarding the thermal stability, oxidation, and combustion of compounds and in case of stoichiometric reaction processes it reveals the molecular mass of the split compound. Furthermore, evolved gas analysis by MS coupled to TGA enables identification of gasseous compounds during combustion.

Charred residues after pyrolysis were used by others to determine the chemical composition by considering the initial polymer stoichiometry [29]. Wilson *et al.*, e.g., used that approach and determined the composition by studying the formation of oxycarbides during heating under inert atmosphere of different polysiloxane samples by TGA [30]. Rachini *et al.*, utilized the weight loss from 150 up to 380 °C of TGA for quantification of 3–aminopropylsilane grafted onto hemp fibers [20]. Similar thermal properties of the treated and untreated cellulose, however, hamper a precise differentiation of single decomposition steps. This restricts TGA measurements to indirect conclusions via comparative determinations and calculations—the accuracy, however, is limited.

In this work, we demonstrate a new method for quantification of immobilized silane groups on cellulose combining both, the charred residue determined by TGA and weight ratios of C, O, H, and Si atoms determined by EA. Therefore, silylation of microcrystalline cellulose was carried out with different concentrations of (3–mercaptopropyl)trimethoxysilane (MRPS) and appropriate functional groups present on cellulose surface were identified by FT-IR spectroscopy and solid state NMR. Additionally, polarization microscopy and solid state NMR were utilized to study the impact of chemical treatment on cellulose crystallinity. Coupling TGA to a mass spectrometer gave further information on the products formed during pyrolysis.

The main advantages of this new approach is that only one calibration is necessary for routine analyses of additional samples and TGA-MS coupling gives further information on thermal stability and pyrolysis processes.

## 2. Results and Discussion

### 2.1. Surface Modification of Microcrystalline Cellulose

**Scheme 1:**
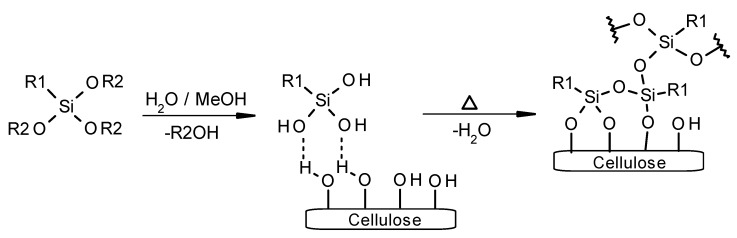
Hydrolysis of alkoxysilane (R1: organic functionality; R2: methyl, ethyl), adsorption onto a cellulose surface of corresponding silanol derivative R1Si(OH)_3_ and subsequent condensation with cellulosic hydroxyl groups and self-condensation.

The surface of microcrystalline cellulose (MCC) was modified by chemical treatment with an alkoxysilane. Chemisorption of silanes on cellulosic surfaces was described as a three step process starting firstly with the hydrolysis of alkoxysilanes yielding reactive silanol groups (Scheme 1). Secondly, silanol groups adsorb physically on the surface of MCC [31]. A subsequent covalent linkage can be achieved by a condensation reaction of silanol groups and hydroxyl groups on cellulosic surfaces at elevated temperatures around 100 °C representing the chemisorption as third step [32]. Aside a condensation reaction of cellulose hydroxyl and silanol groups a homopolycondensation of alkoxysilanes or corresponding silanol derivatives takes place yielding a polysiloxane layer [33].

In this contribution (3–mercaptopropyl)trimethoxysilane (MRPS) was used to surface modify MCC in presence of triethylamine as a base. MRPS generates thiol functionalities on the surface of cellulose, which can be used for further coupling reactions such as thiol-ene chemistry [34]. MCC was treated with six equidistant concentrations of MRPS ranging from 0.27 up to 1.6 mmol/L yielding cellulose samples with six different degrees of functionalization (Cel–1 to Cel–6) in comparison to the neat microcrystalline cellulose (MCC) (Table 1). Additionally, polyMRPS was prepared as reference by homopolycondensation MRPS in absence of cellulose. Soxhlet extractions were performed in ethanol for 16 h after silane treatment in order to remove all physisorbed silane fractions.

### 2.2. Characterization of Surface Modified Cellulose

In order to gain insight into the silylation process the modified samples were characterized with respect to the presence of functional groups by FT-IR and Raman spectroscopy. Additionally, the impact of surface modification on morphology and the binding state was studied by polarized microscopy and solid state NMR.

#### 2.2.1. Analysis of Functional Groups by FT-IR and Raman Spectroscopy

**Figure 1:**
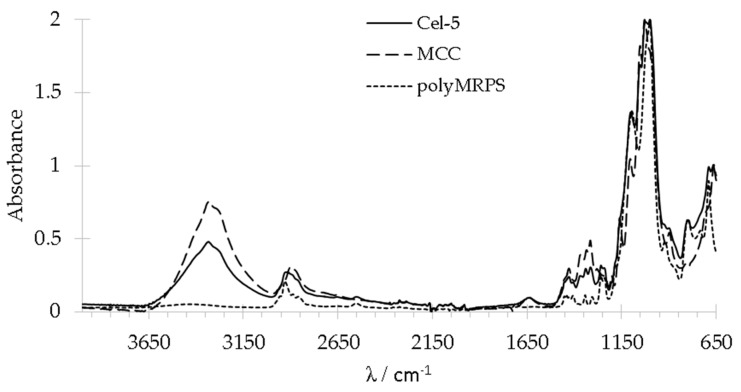
FT-IR spectra of neat cellulose (MCC), cellulose treated with 1.35 mmol/L MRPS (Cel–5) and polyMRPS.

**Figure 2:**
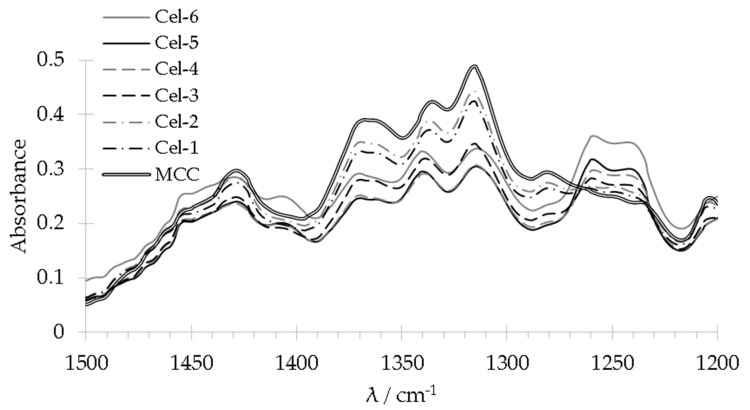
Enlarged section of Si–C stretching 1240 and 1260 cm^−1^ for comparing the degree of cellulose functionalization of the samples Cel–1 to Cel–6.

All silylated cellulose and reference samples were studied with FT-IR spectroscopy after appropriate purification (Figure 1). PolyMRPS and MRPS treated cellulose Cel–5 showed signals belonging to symmetrical stretching of S–H bonds at 2551 cm^−1^, silicon bonds at 1240 and 1260 cm^−1^ as well as to symmetric and asymmetric stretching of ν_s_ Si–O–Si and ν_as_ Si–O–C bonds at 690 and 800 cm^−1^. Signals at 2930 cm^−1^ as well as 1455 and 1306 cm^−1^, which were assigned to C–H stretching and CH_2_ in plane and out of plane scissoring, respectively and signals between 1184 and 650 cm^−1^ (Si–OH, ν_as_ Si–C, ν_as_ Si–O–Si) overlapped partially with C–H, C–O, and C–C stretching belonging to cellulose. Signals at 1642 cm^−1^ and at 3330 cm^−1^ were attributed to O–H stretching at cellulose and to adsorbed water, respectively. For an initial estimation of functionalization degree, signals for silicon bonds at 1240 and 1260 cm^−1^ (ν Si–C) were utilized. Figure 2 depicts the FT-IR spectra of Cel–6 to Cel–1 ranging from 1500 up to 1200 cm^−1^. The intensity of the stretching signal ν Si–C increases with increasing MRPS concentration used for surface modification. Thus, treatment with different MRPS concentrations yielded cellulose samples with low and high amount of silane groups on MCC surface.

**Figure 3:**
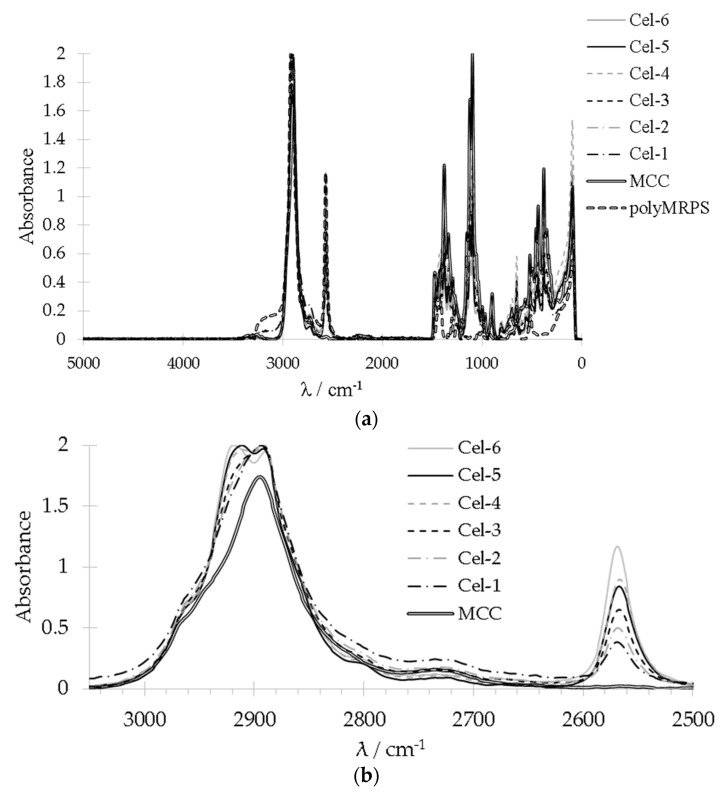
(**a**) Overview and (**b**) enlarged sections of Raman spectra of samples Cel–1 to Cel–6.

Due to the weak infrared sensitivity of thiols in FT-IR all MRPS modified cellulose samples were studied additionally with Raman spectroscopy. The overview spectra of the references MCC, polyMRPS, and samples Cel–1 to 5 as well as the enlarged sections showing the CH_2_ signal at 2900 cm^−1^ and a signal at 2568 cm^−1^ corresponding to the S–H stretching of thiol groups are depicted in Figure 3a,b. That signal showed a steadily increase in intensitiy from Cel–1 treated with the lowest up to Cel–6 treated with the highest MRPS concentration except for sample Cel–5, which exhibited a lower stretching intensitiy than expected. Probably a partial thiol oxidation during storage at ambient conditions caused that slight deviation. Raman spectroscopy revealed that thiol groups are available on the cellulose surface after silylation of MCC with MRPS.

#### 2.2.2. Morphology and Binding State

As material properties strongly depend on the chemical and physical constitution, the morphology as well as the binding state of the treated samples were investigated using polarized light microscopy and solid state NMR.

**Figure 4:**
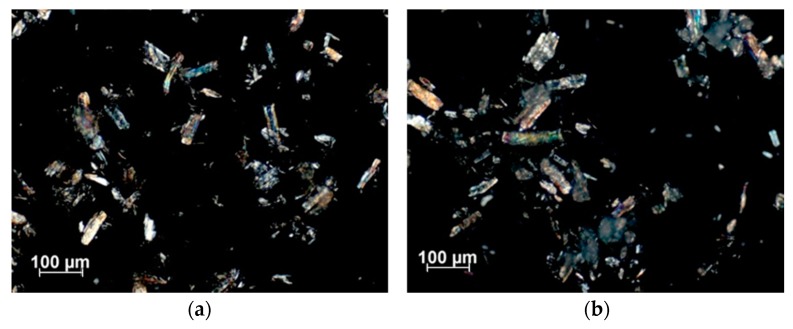
10 wt % of (**a**) native cellulose (MCC); and (**b**) Cel–5 in water recorded with a 5-fold magnification and with an angle of 170°.

The following polarized microscopy images show two aqueous mixtures each of them containing 10 wt % of native MCC as well as Cel–5, respectively (Figure 4a,b). For both, the native and modified cellulose, birefringence occur before and after silylation. Obviously, the crystallinity of MCC was maintained after silane treatment.

**Figure 5:**
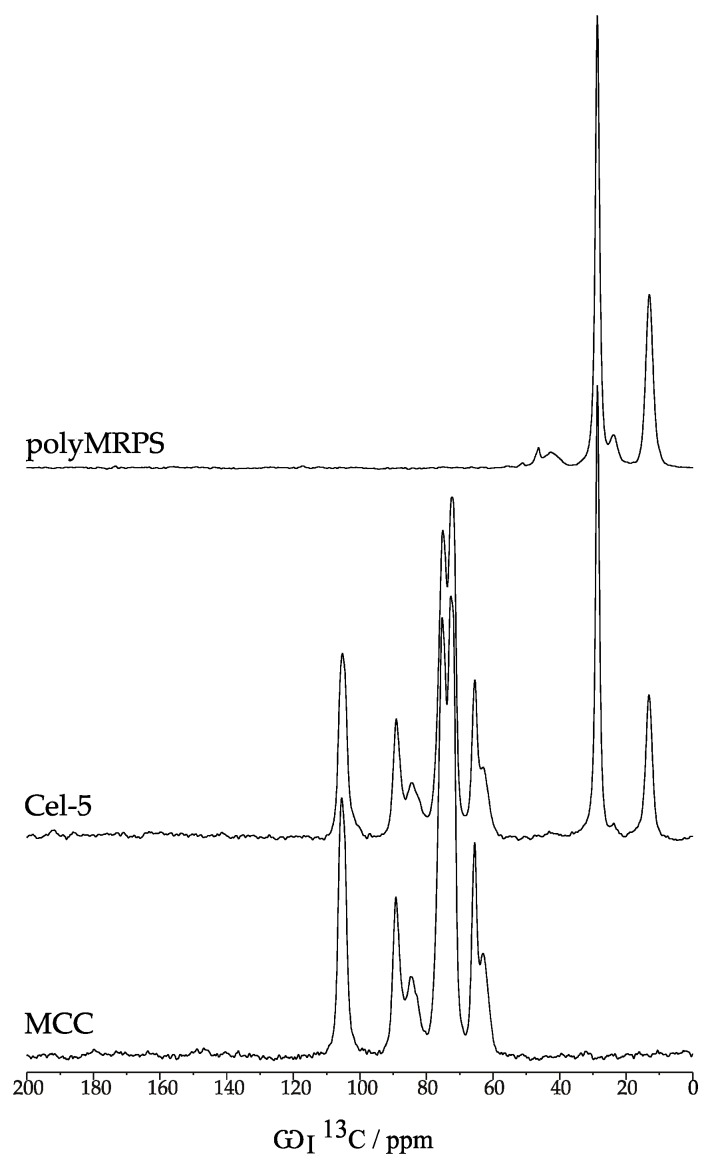
Solid state cross polarization magic angle spinning (CP-MAS) ^13^C NMR spectra of polyMRPS (**top**); Cel–5 (**middle**); and MCC (**bottom**). All spectra were recorded under the same conditions (see Section 3).

**Figure 6:**
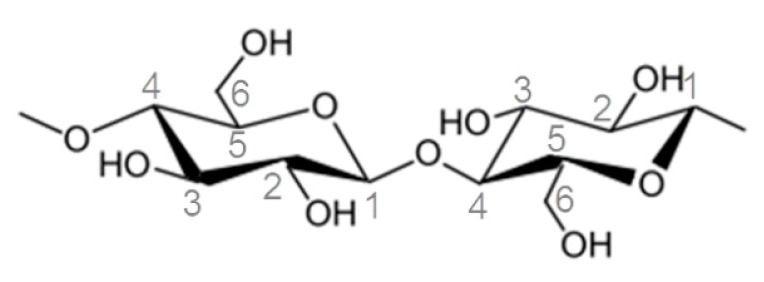
Chemical structure of a cellobiose unit in cellulose samples.

Furthermore, solid state ^13^C NMR spectroscopy was performed in order to gain additional information about crystalline and amorphous morphology as well as the type of linkage between cellulose and MRPS (Figure 5, top: polyMRPS, middle: silylated cellulose Cel–5 and bottom: native cellulose). The ^13^C chemical shifts at 105.4, 89.2, 84.5, 75.2, 72.6, 65.4, and 62.9 ppm were attributed to the appropriate C-atoms and it was distinguished between amorphous (A) and crystalline (C) fractions (Figure 6, Table 2) [35,36]. The ratio of amorphous and crystalline fractions in a non-treated MCC sample was similar to the one in silane treated Cel–5 indicating that crystallinity was maintained during silylation. This is in agreement with the observations of appropriate birefringence by polarized light microscopy. The signals at 28.6 and 13.2 ppm were attributed to the propyl chain of the silane (polyMRPS and Cel–5) confirming the presence of silane at the cellulose surface. Basically, the hydroxy groups are mainly responsible for the reaction of cellulose. However, the reactivity of the primary alcohol group at position C–6 was proven to react faster in comparison to the secondary OH groups at C–2 and C–3 in different chemical reactions [37]. Thus, the condensation reaction of silanol groups was expected to take place in the first place at OH–C–6 causing a shift in the C–6 signal towards 70 ppm. However, no changes in signals for C–6, –3, and –2 were observed comparing the chemical shifts of MCC and Cel–5.

### 2.3. Thermal Properties and Silane Loading Determination

FT-IR and Raman spectroscopy enabled identification of functional groups and a qualitative estimation of the degree of functionalization after treating microcrystalline cellulose with different concentrations of MRPS. In the following section a TGA-MS was utilized in order to study thermal behavior and gas evolution during the pyrolysis process. Correlated with elemental analysis, TGA-MS will be discussed as a method for quantification of the grafted amount of silane on microcrystalline cellulose.

#### 2.3.1. Studying Thermal Behavior with Thermogravimetric Analyses

**Figure 7:**
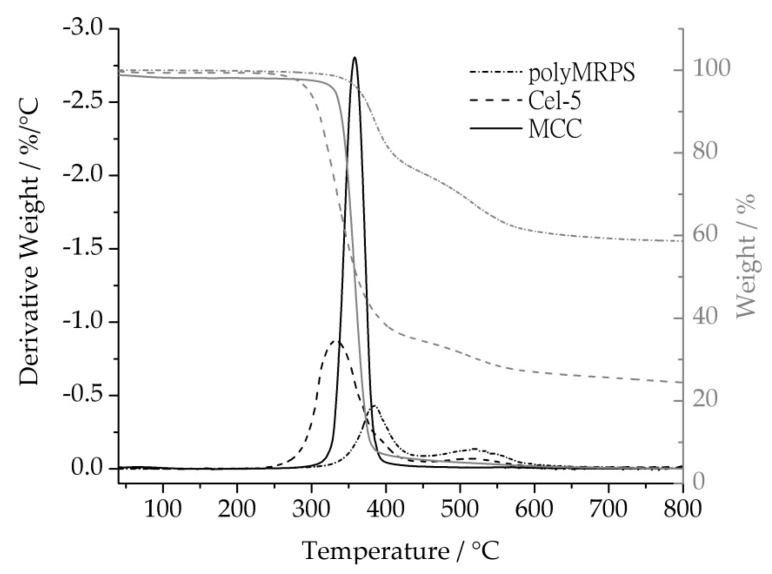
Mass loss curves and first derivative of a mass loss curve comparing native cellulose, silylated cellulose (Cel–5), and the polysiloxane polyMRPS.

In the first instance, TGA measurements were performed to evaluate the thermal properties of surface modified cellulose by comparing the maxima of the first derivative of the weight loss curves and the charred residues. Generally, cellulose shows initial decomposition in one step ranging from 300 to 400 °C. Polysiloxanes were described to decompose in two steps at a lower temperature range of 350–450 °C and 450–550 °C. Figure 7 displays the first derivative of Cel–5 as silane modified cellulose in comparison to native cellulose and polyMRPS as references. The neat cellulose sample MCC showed a one step weight loss with a maximum at 358 °C (63 min) and with a preliminary slight decrease in weight at 100 °C due to loss of water (1.5%–3%). The polysiloxane reference polyMRPS decomposed in two steps showing maxima at 396 °C (67 min) and at 525 °C (80 min). The degradation of Cel–5 was observed between 250 °C and 550 °C with maximum rates of weight loss at the temperatures 331 °C with a downstream shoulder at 394 °C (67 min) and 512 °C (79 min). The first step represents cellulose decomposition whereas the two additional steps in the derivative of weight loss were attributed to siloxane pyrolysis. The reduction in weight loss temperature of Cel–5 in comparison to the neat cellulose suggests a slight reduction in thermal stability of surface silylated cellulose.

#### 2.3.2. Anaylsis of Evolved Gas During Heating by MS

**Figure 8:**
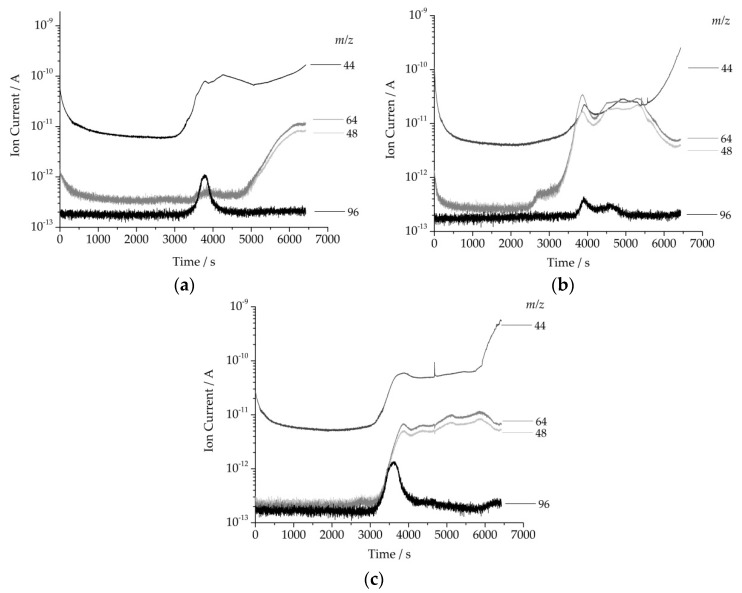
Multi-ion-detector mass spectrum displaying mass to charge ratios (*m*/*z* = 44, 48, 64, 96) of (**a**) native cellulose; (**b**) polyMRPS; and (**c**) Cel–5.

In order to gain insight into the degredation process the evolved gas during heating was analyzed with a coupled quadrupole mass spectrometer (Figure 8). Generally, cellulose evolves furan derivates e.g., furfural (*m*/*z* = 96) during decomposition under helium atmosphere [38]. Furthermore, CO_2_ (*m*/*z* = 44) is generated due to the appropriate oxygen containing functional groups (–OH, –O–) of cellulose causing oxidation processes. Sulfur is present in a sample, e.g., thiols SO_2_ (*m*/*z* = 64) is being formed as oxidation product and SO (*m*/*z* = 48) as a fragment [39]. The multi-ion detection (MID) mass spectrum of cellulose revealed the presence of *m*/*z* = 44 and 96 showing a maximum around 360 °C (63 min), which was in good agreement with the temperature of maximum rate of weight loss. In contrast to this, the MID of polyMRPS exhibited a maximum approximately at 380 °C (65 min) for the masses *m*/*z* = 48 and 64 for SO and SO_2_, respectively. This indicated a correlation to the maximum at 396 °C of the rate of weight loss in TGA. The mass to charge ratios *m*/*z* = 44, 48, 64, and 96 were found for Cel–5 with their maximum intensities around 330 °C (60 min) and 380 °C (65 min). The formation of SO, SO_2_, and furfural demonstrated the presence of thiol functionalities and therefore a siloxane layer onto cellulose surface.

Furthermore, charred residues were studied in order to estimate the thermal stability in addition to initial decomposition temperatures and to determine the amount of grafted silane onto cellulose surface. Polysiloxanes were described to form silicon oxycarbide as charred residue during heating under inert gas [30], which makes them feature excellent thermal behavior. Thus, polyMRPS and Cel–5 showed high charred residues of 65% and 25%, respectively, whereas the native cellulose decomposed almost completely. Hence, the initial decomposition of Cel–5 started at slightly lower temperatures compared to native cellulose. The higher charred residue, however, improved the thermal stability of surface modified cellulose, which is attributed to silicon oxycarbide formation.

#### 2.3.3. Determination of Silane Loading by TGA and EA Correlation

**Figure 9:**
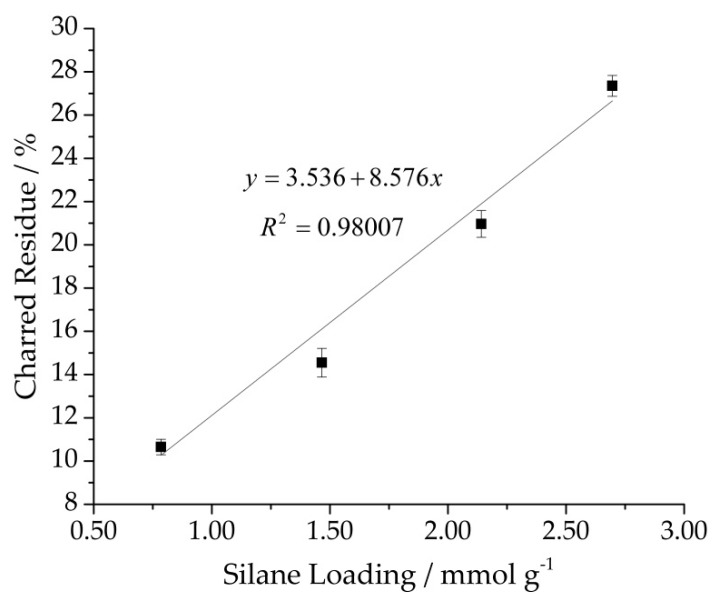
Charred residue determined by TGA as function of silane loading determined with elemental analysis.

As the charred residue is proportional to the quantity of grafted silane, TGA measurements were applied to determine silane content of the silylated cellulose samples quantitatively. Elemental analysis (EA) was used as reference values and correlated with charred residues. Therefore, modified cellulose samples Cel–1 to Cel–6 were analyzed by EA. From the determined mass ratios for C, O, H, and Si appropriate amount of MRPS onto cellulose in mmol/g was calculated. With increasing initial concentration of MRPS, the charred residue as well as the silane loading determined by EA increase linearly yielding 2.696 mmol MRPS per gram cellulose. As cellulose tends to adsorb water, weight losses between 150 and 800 °C were taken into account for quantification. The charred residue as a function of silane loading determined by EA resulted in a linear dependency with a correlation coefficient (*R*^2^) of 0.98 (Figure 9). Thereby, further silylations were analyzed with the help of the linear dependency (Figure 9) and yielded samples with a silane loading ranging from 0.8 up to 3.1 mmol/g (Table 3). The samples that were analyzed by TGA and EA function showed only a slight deviation in comparison to the loading determined directly with EA.

In order to verify our results, we have calculated the MRPS loading using a calculation approach described by Rachini *et al*. This method uses the difference in weight loss during TGA measurement from 150 up to 380 °C comparing native and modified cellulose (Equation (1)) [20]. MRPS loading for sample Cel–5 was calculated with that approach using Equation (1) where $\left|W_{150-380}\right|$ represents the difference in weight loss (%) between native and modified cellulose and M the molar mass of a hydrolyzed silane (M_hydrol. MRPS_ = 154.29 g/mol).

(1) $$\text{silane loading}\;\left(\text{mmol}/g\right)=\frac{\left|W_{150-380}\right|\times 1000}{M}$$

With a silane loading of 2.1 mmol/g for Cel–5, this approach gave lower values related to elemental analysis and TGA/EA correlation. Most likely, overlapping of the appropriate decomposition steps during heating and therefore making determination of the accurate difference in weight loss causes the deviation difficult.

With the help of TGA/EA correlation, it is possible to convey problems of process overlapping and to get reliable values regarding the degree of functionalization. Based on the linear dependency of TGA and EA, further samples can easily be characterized by TGA with respect to both their thermal properties as well as their composition.

## 3. Materials and Methods

All chemicals were used as received from commercial suppliers without further purification. The microcrystalline cellulose (MCC), named Avicel PH-101^®^, was provided by FMC BioPolymer (Sandvika, Norway). The average particle size of Avicel PH-101^®^ was about 50 µm. The silane coupling agent (3–mercaptopropyl)trimethoxysilane (MRPS) was purchased from Sigma-Aldrich (95%, Steinheim, Germany). All reactions with alkoxysilane were carried out in deionized water. Triethylamine (TEA) was obtained from Merck (99%, Darmstadt, Germany). Methyl ethyl ketone, acetone, and ethanol were of technical grade.

All reactions were carried out under nitrogen atmosphere and magnetic stirring in a 100 mL reaction flask equipped with a reflux condenser.

### 3.1. Characterization

#### 3.1.1. Fourier Transformed Infrared Spectroscopy (FT-IR)

FT-IR spectra were collected on an Equinox 55 FTIR spectrometer (Bruker Optic GmbH, Ettlingen, Germany) equipped with a horizontal Golden Gate cell in attenuated total reflection (ATR) mode. All spectra were recorded from 4000 to 650 cm^−1^ with a resolution of 4 cm^−1^ and 32 scans. For data evaluation, the software OPUS 5.5 (Bruker Optic GmbH, Ettlingen, Germany) was applied.

#### 3.1.2. Raman Spectroscopy

Raman spectroscopy was performed on a Vertex 80 spectrometer (Bruker Optic GmbH, Ettlingen, Germany) equipped with an excitation wavelength of 1064 nm. All spectra were recorded from 5000 up to 0 cm^−1^ with a resolution of 4 cm^−1^ and 250 scans. The data were evaluated using the software OPUS 5.5 (Bruker Optic GmbH, Ettlingen, Germany).

#### 3.1.3. Elemental Analysis

EA was performed by Mikroanalytisches Labor Pascher (Remagen, Germany). The elements C, H, O, S, und Si were examined.

#### 3.1.4. Thermogravimetric Analysis Coupled with a Quadrupol Mass Spectrometer (TGA-MS)

The pyrolysis of silylated cellulose was carried out using a TGA Q5000 V3.15 Build 263 (TA Instruments, Eschborn, Germany) coupled with an OmniStar (Pfeiffer Vakuum, Aßlar, Germany) quadrupole MS. All MS related measurements were performed under helium atmosphere (99.996%, Linde Gas, Munich, Germany). The flow rate of helium was 40 mL/min. The samples (between 2 and 3 mg) were placed in a platinum pan. The system was equilibrated first with helium for 30 min at ambient temperature, then the sample was heated with a rate of 10 °C/min from ambient temperature up to 800 °C. Multiple ion detection (MID)-MS spectra (*m*/*z* = 44, 48, 64 and 96) of the combustion gas were recorded during the pyrolysis of the samples. The TGA and MS data were evaluated with the software TA Advantage v.5.5.3 (TA Instruments, Eschborn, Germany) and Quadera 4.50 (Pfeiffer Vakuum, Aßlar, Germany), respectively.

#### 3.1.5. Solid State CP-MAS ^13^C NMR Spectroscopy

Solid state CP-MAS ^13^C NMR spectra were collected on a Bruker-Avance spectrometer with a 14.07 T magnet, operating at ^1^H and ^13^C Larmor frequencies of 600.13 MHz and 150.903 MHz, respectively (Bruker Biospin, Karlsruhe, Germany). A 4 mm triple resonance magic angle spinning (MAS) probe using a MAS frequency of 13.333 kHz and VT-set temperature of 270 K (corresponding to 294 K sample temperature) was used. The ^13^C cross polarization (CP) experiments were done using the following experimental conditions optimized with unlabeled glycine: (1) ^1^H excitation pulse 3.15 µs; (2) Magnetization transfer from ^1^H to ^13^C via a ramped CP with a contact time of 2 ms. Spinlock field strengths of 59 kHz and 71 kHz were used for ^1^H and ^13^C, respectively [40]; (3) During acquisition, proton decoupling using small phase incremental alternation with 64 steps (SPINAL 64) using a proton radio frequency (RF) field of 90 kHz was applied [41]. Each spectrum was recorded with 1024 scans. The recycle delay between each scan was set to 3 s. The ^13^C chemical shift was referenced indirectly using the adamantane peaks (CH = 29.5 ppm and CH_2_ = 38.56 ppm).

#### 3.1.6. Polarized Light Optical Microscopy

Images were recorded on a Zeiss Axio Imager.M1 (Carl Zeiss Microscopy GmbH, Jena, Germany) polarized optical microscope equipped with a digital camera (AxioCAM MRc., Hamburg, Germany).

### 3.2. Chemical Modification

#### Silylation of Microcrystalline Cellulose

The silylations of microcrystalline cellulose (2.5 g) were carried out with different amounts of (3–mercaptopropyl)trimethoxysilane (MRPS) ranging from 2.7 to 16.2 mmol in 10 mL water. In case of base catalysis 0.1 equivalents of triethylamine were added to the mixture. The suspension was stirred and heated at 120 °C for 1.5 h. After cooling to ambient temperature, the solid was filtered off and washed with deionized water, acetone, and methyl ethyl ketone. A fine white powder was obtained. The raw products were purified by Soxhlet extraction in 150 mL ethanol for 16 h and dried afterwards for 72 h under ambient conditions.

## 4. Conclusions

In this work, we demonstrate a new method for quantification of silane immobilized on cellulose combining both TGA and EA. Therefore, microcrystalline cellulose was treated with (3–mercaptopropyl)trimethoxysilane (MRPS) in basic aqueous solution. Surface modified celluloses were studied by FT-IR, Raman, solid state NMR spectroscopy, and polarized light microscopy in order to determine functional groups present on the surface and to study the impact of chemical treatment on cellulose crystallinity. Information on thermal stability and the pyrolysis process was gained by thermogravimetric analysis with a coupled mass spectrometer. With respect to thiol functionalized cellulose samples, furfural (*m*/*z* = 96) as well as SO_2_ (*m*/*z* = 64) and SO (*m*/*z* = 48) were detected in the decomposition gas at a time and temperature scale that was in a good agreement with the weight losses in TGA. In order to determine the exact silane loading onto cellulose surface, the mass percentages of the appropriate elements were quantified by elemental analysis and correlated with the charred residues determined by TGA. A linear correlation was obtained by plotting the charred residue from TGA as a function of silane loading from EA. According to this correlation, it is possible to determine silane loading for additional samples utilizing just TGA.

In summary, TGA-MS as a facile and fast method was utilized for a quantitative analysis after an appropriate calibration as well as a qualitative investigation of the pyrolysis. The main advantage of this new approach is that only one calibration is necessary for routine analyses of additional samples. TGA-MS coupling gives further information on thermal stability and pyrolysis routes.

## Figures and Tables

**Table 1:** Surface modification of microcrystalline cellulose with various initial MPRS concentrations.

Sample	Cel–1	Cel–2	Cel–3	Cel–4	Cel–5	Cel–6
[MRPS]_0_ (mol/L)	0.27	0.54	0.81	1.08	1.35	1.62

**Table 2:** Assignment of chemical shifts in ppm to carbon atoms and distinction of amorphous (A) and crystalline (C) fractions in cellulose samples.

Sample	C–1	C–4	C–4	C–2,3,5	C–2,3,4	C–6	C–6	–CH_2_–S	Si–CH_2_
		C	A	A	C	C	A		–CH_2_–
polyMRPS	–	–	–	–	–	–	–	28.6	13.2
Cel–5 ^1^	105.4	89.2	84.5	75.2	72.6	65.4	62.9	28.6	13.2
MCC	105.4	89.2	84.5	75.2	72.6	65.4	62.9	–	–

^1^ purified by Soxhlet extraction in ethanol.

**Table 3:** TGA and EA analyses of surface modified cellulose samples with MRPS.

Sample	Cel–1	Cel–2	Cel–3	Cel–4	Cel–5	Cel–6
[MRPS]_0_ (mol/L)	0.27	0.54	0.81	1.08	1.35	1.62
^1^ charred residue (%)	10.7 ± 0.4	14.6 ± 0.7	21.0 ± 0.6	24.0 ± 0.5	27.4 ± 0.5	30.3 ± 0.8
^2^ MRPS loading (exp.) (mmol/g)	0.785	1.466	2.141	–	2.696	–
^3^ MRPS loading (calc.) (mmol/g)	0.83 ± 0.04	1.28 ± 0.08	2.03 ± 0.07	2.38 ± 0.05	2.78 ± 0.6	3.12 ± 0.1

^1^ charred residue of samples purified by soxhlet extraction determined in the temperature range of 150 up to 800 °C with *n* = 3; ^2^ determined by elemental analysis; ^3^ determined by calculation with Equation (1).

## Supplementary Materials

The following are available online at www.mdpi.com/1996-1944/9/6/415/s1. Figure S1: TGA measurements of Cel–1 with *n* = 3; Figure S2: TGA measurements of Cel–2 with *n* = 3; Figure S3: TGA measurements of Cel–3 with *n* = 3; Figure S4: TGA measurements of Cel–4 with *n* = 3; Figure S5: TGA measurements of Cel–5 with *n* = 3; Figure S6: TGA measurements of Cel–6 with *n* = 3; Figure S7: Overview FT-IR spectrum of the references MCC and polyMRPS and silane modified samples Cel–1 to Cel–6; Table S1: Elemental analysis values.

## Abbreviations

The following abbreviations are used in this manuscript:

EA	Elemental analysis
CP-MAS	Cross polarization magic angle spinning
FT-IR	Fourier transform infrared spectroscopy
MCC	Microcrystalline cellulose
MID	Multi ion detector
MRPS	(3–mercaptopropyl)trimethoxysilane
RF	Radio frequency
SPINAL 64	Small phase incremental alternation with 64 steps
TGA-MS	Thermogravimetric analysis coupled with a mass spectrometer
