# Supplementary material for: Quantitative and Qualitative Analysis of Surface Modified Cellulose Utilizing TGA-MS
Source: Materials (Basel). 2016 May 25;9(6):415. doi: 10.3390/ma9060415 (PMC5456835; doi:10.3390/ma9060415)
Supplement: Supplementary file 1 [file materials-09-00415-s001.pdf]

Supplementary Materials: Quantitative and Qualitative Analysis of Surface Modified Cellulose Utilizing TGA-MS

Daniel Loof, Matthias Hiller, Hartmut Oschkinat and Katharina Koschek

1. Thermogravimetric Analyses of All Silane Modified Cellulose Samples

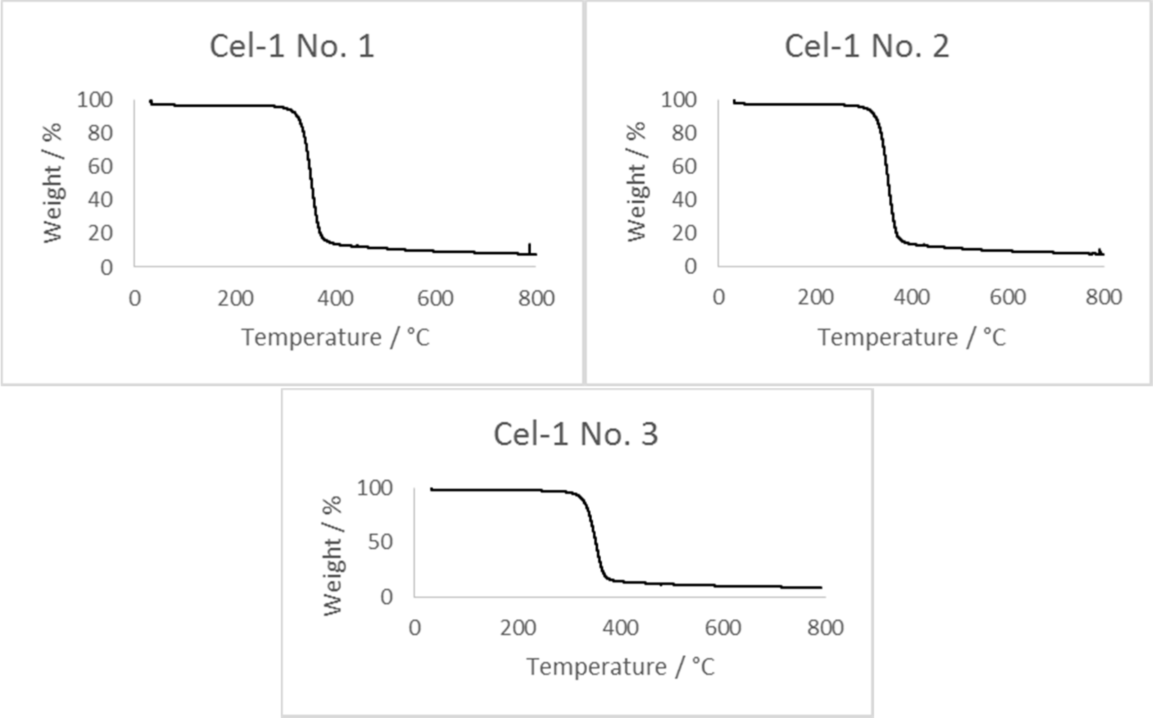

Figure S1. TGA measurements of Cel–1 with n = 3.

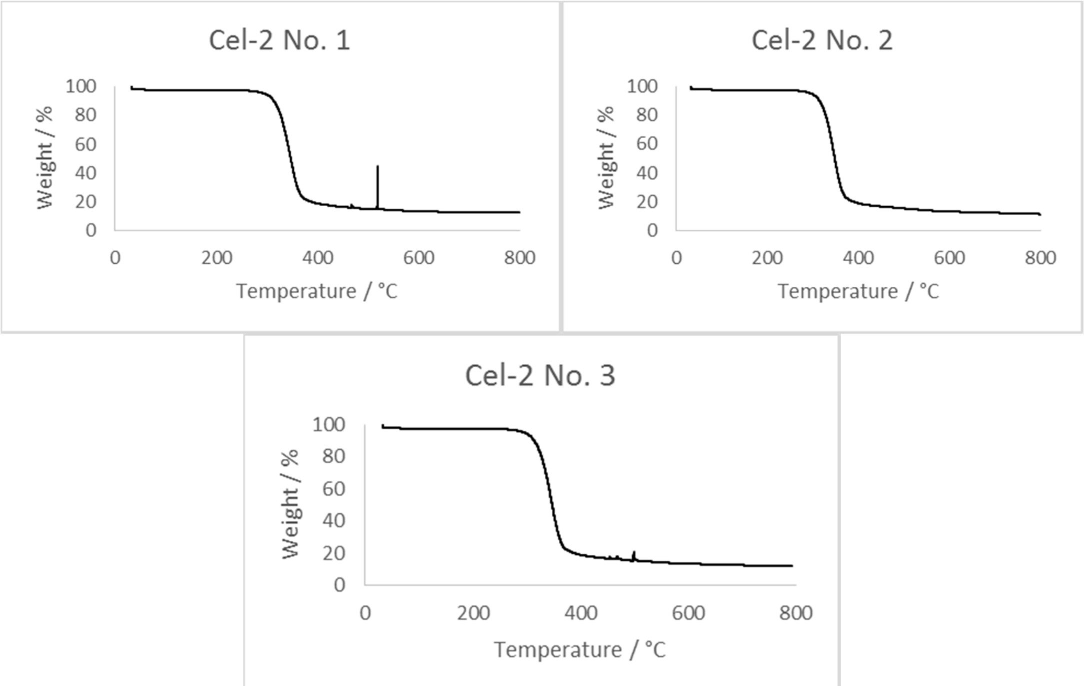

Figure S2. TGA measurements of Cel–2 with n = 3.

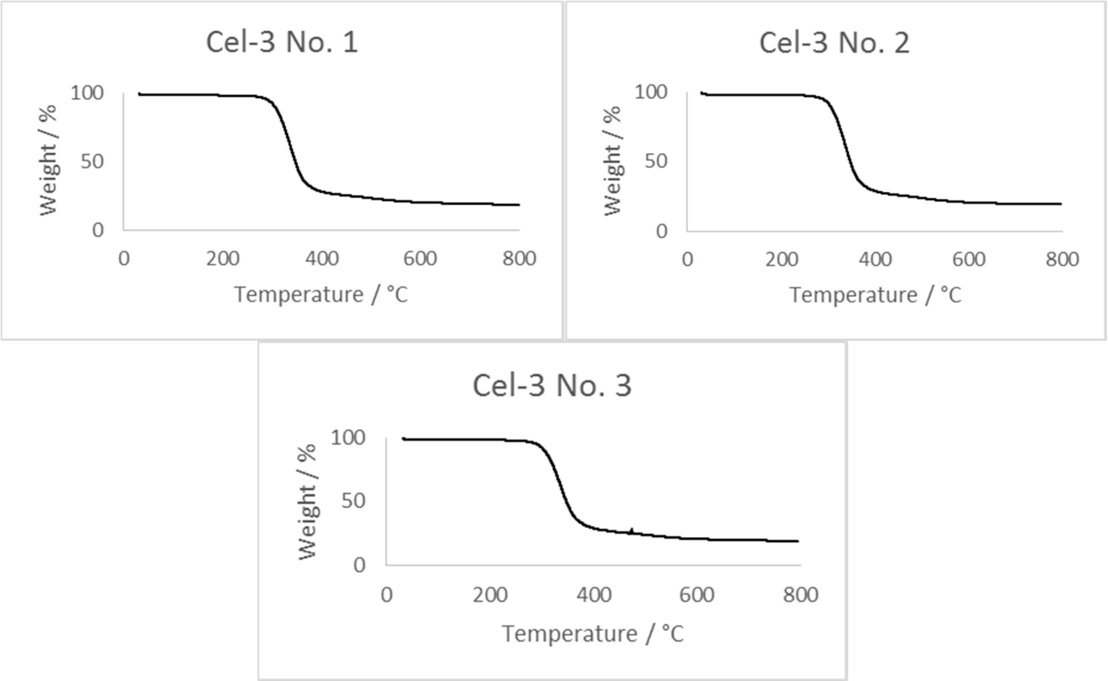

Figure S3. TGA measurement Cel–3 with $n = 3$.

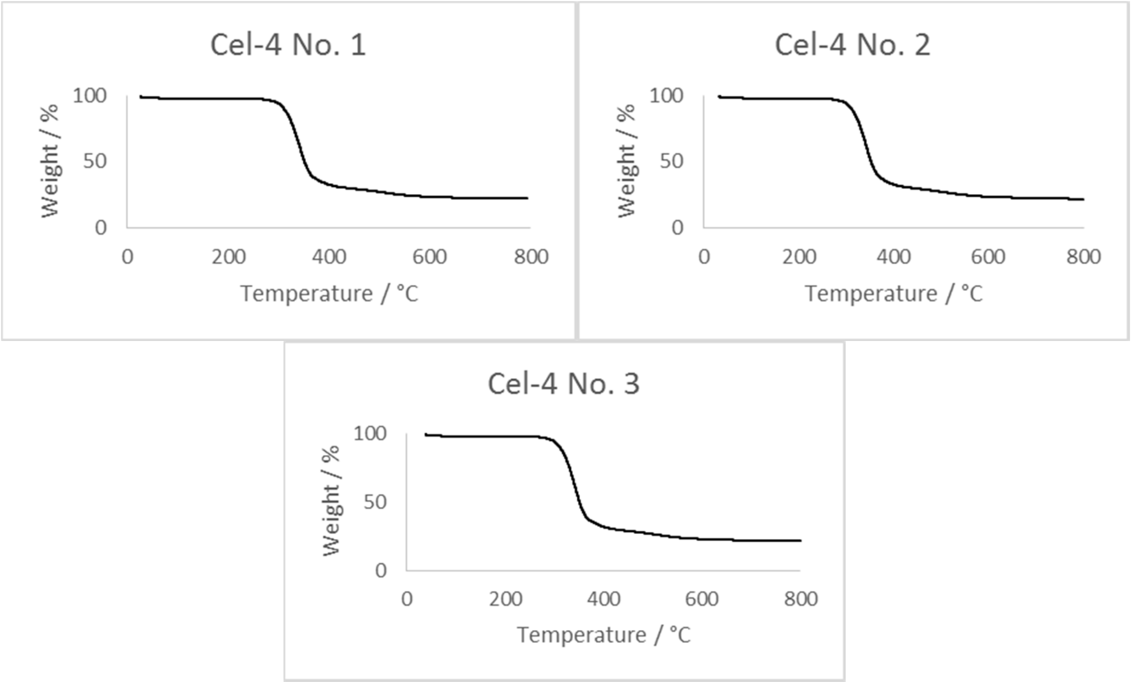

Figure S4. TGA measurement of Cel–4 with $n = 3$.

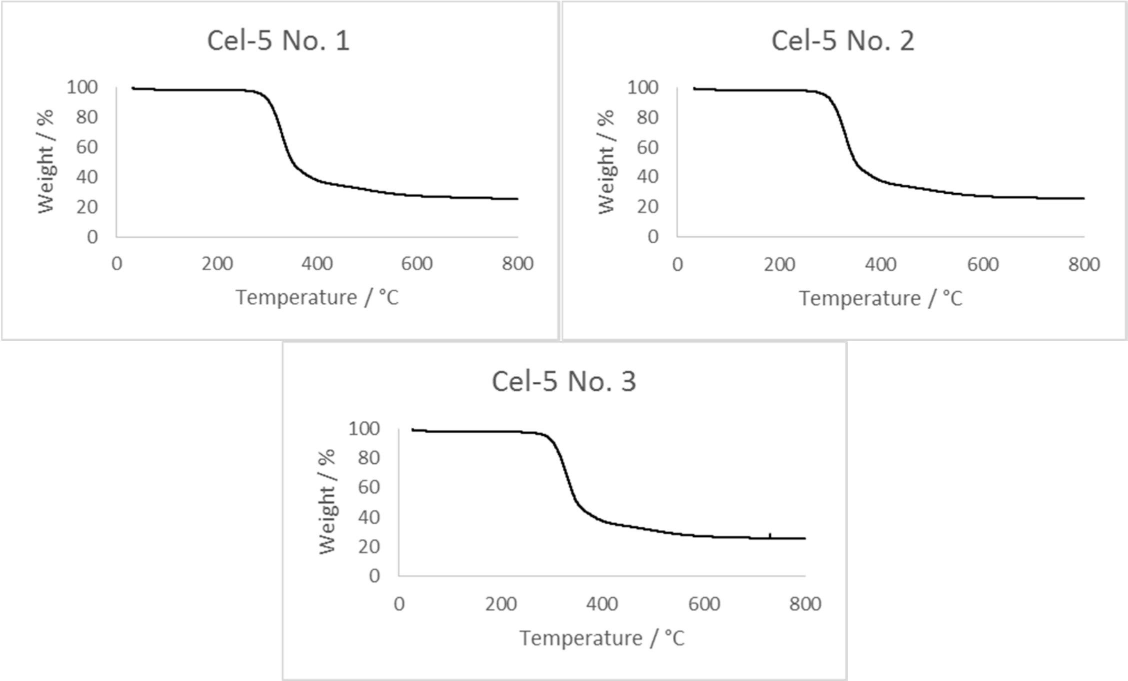

Figure S5. TGA measurement of Cel–5 with *n* = 3.

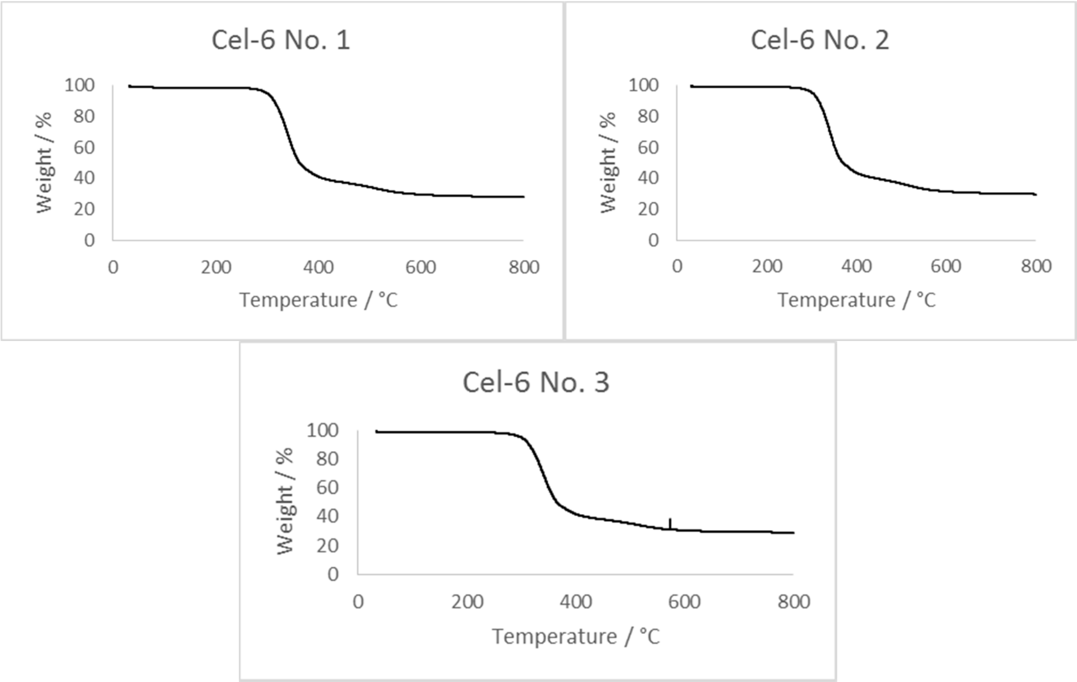

Figure S6. TGA measurement of Cel–6 with *n* = 3.

2. FT-IR and Elemental Analyses

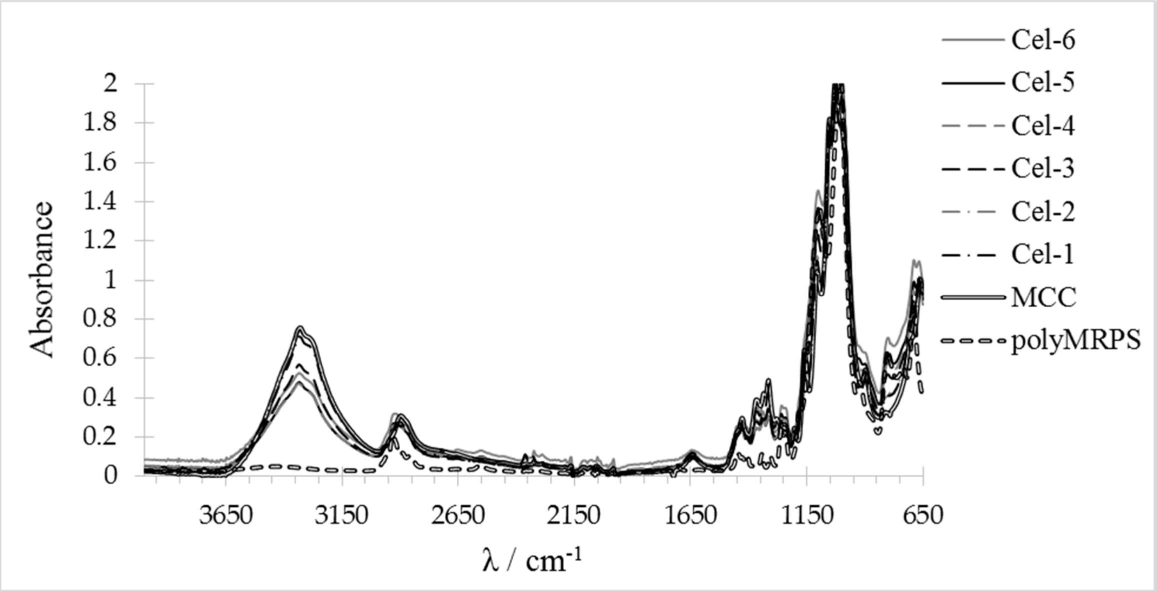

Figure S7. Overview FT-IR spectrum of the references MCC and polyMRPS and silane modified samples Cel–1 to Cel–6.

Table S1. Elemental Analysis Values.

	M/g mol⁻¹	Cel–1	Cel–2	Cel–3	Cel–4	
**C**	12.0107	39.39	37.92	36.4	35.57	wt %
**H**	1.00794	6.46	6.4	6.31	6.14	wt %
**O**	15.9994	48.4	43.5	41.9	38.3	wt %
**S**	32.065	2.66	4.7	6.87	8.68	wt %
**Si**	28.0855	2.08	4.12	6.01	7.54	wt %
Σ		98.99	96.64	97.49	96.23	wt %
**S**	32.065	0.830	1.466	2.143	2.707	mmol/g
**Si**	28.0855	0.741	1.467	2.140	2.685	mmol/g
Σ(Si + S)/2		0.785	1.466	2.141	2.696	mmol/g
